# Discrimination and intimate partner violence among a sample of bisexual and gay men in the United States: a cross-sectional study

**DOI:** 10.3389/fpubh.2023.1182263

**Published:** 2023-07-31

**Authors:** Khyati Rustagi, Luzan JadKarim, Nick Birk, Alvin Tran

**Affiliations:** ^1^Department of Population Health and Leadership, University of New Haven, West Haven, CT, United States; ^2^Harvard T.H. Chan School of Public Health, Boston, MA, United States

**Keywords:** domestic violence, intimate partner violence, gay men, bisexual men, sexual minority men, perceived discrimination, minority stress

## Abstract

**Purpose:**

Intimate partner violence (IPV) is becoming more recognized as a public health concern among sexual minority men, including bisexual and gay men. Guided by the Minority Stress Model, we assessed the relationship between perceived discrimination and three forms of IPV among a sample of bisexual and gay men living in the United States.

**Methods:**

We analyzed data as part of the Men’s Body Project, a cross-sectional study launched in 2020 to assess health behaviors of bisexual and gay men.

**Results:**

A total of 549 individuals participated in the survey, of which 52% were gay and 48% were bisexual men. Perceived discrimination was significantly associated with elevated odds ratios ranging from 1.15 to 1.18 across three forms of IPV, with Physical IPV odds ratio being highest.

**Conclusion:**

Given the significant association between perceived discrimination and IPV, interventions aimed at addressing IPV experiences among sexual minority men must consider the role of minority stress.

## 1. Introduction

Intimate partner violence (IPV) is a global health concern. While it is often perceived as an issue primarily occurring within heterosexual relationships, studies continue to document its prevalence in relationships between sexual minority individuals, including bisexual and gay men (1). IPV refers to any aggressive or abusive behavior occurring in an intimate relationship and may exist in the form of physical violence (e.g., shoving), sexual violence (e.g., forced sexual penetration), stalking (e.g., repeated unwanted calls, texts), and psychological aggression (e.g., humiliation) (2).

In the United States (U.S.), IPV affects an estimated one-third of Americans, including sexual minority men (3). Studies suggest that approximately one out of every four gay men (26.0%) and four out of every ten bisexual men (37.3%) experienced IPV at some point in their lifetime (1). The rate of IPV among bisexual and gay men is often higher than that of their heterosexual male counterparts (1, 4). In particular, estimates from a nationally representative sample show the prevalence of IPV in bisexual, gay, and straight men to be 83, 79, and 64%, respectively (5). Furthermore, many studies have found an equal or higher prevalence of IPV among bisexual and gay men when compared to heterosexual women (4, 6–9).

The number of recent studies assessing the toll of IPV in bisexual and gay male populations is limited, as most have focused on IPV experiences in populations of heterosexual women (2, 7, 10, 11). For example, a systemic review found that up until July 2022, only 78 papers were published on IPV perpetration on PubMed and EBSCO (12). Another review stated that of the estimated 14,200 studies on IPV published between 1999 and 2013, only 400 or 3% examined the issue particularly in bisexual, gay, and lesbian individuals (13). In addition, the negative outcomes of IPV in MSM, such as increased HIV risk due to an increase in condomless anal sex, have not been fully examined in nationally representative studies (14). Even fewer are studies assessing the burden of IPV among bisexual men populations. A report by the National Intimate Partner and Sexual Violence Survey suggests that the rate of physical IPV was highest among bisexual men (15). Most research on IPV has grouped LGB (lesbians, gay, bisexual) as one entity (i.e., sexual minorities), but differences within and between these groups exist (16). For example, bisexual men experience unique stressors (e.g., negative attitudes, microaggression, identity concealment, stigma) that differ from lesbian and gay individuals (17). Due to these unique stressors and discrimination, bisexual men are at elevated risk for a myriad of mental health, substance use, and sexual health problems (18). Hence, it becomes imperative to assess differences in the experiences between bisexual and gay men as they do not represent a homogenous social group.

Few studies have observed how perceived discrimination influences IPV among sexual minority individuals (19, 20). Most studies on IPV have predominantly focused on bisexual and lesbian women (5). In 2013, Edwards and Sylaska documented that discriminatory experiences do not directly lead to same-sex partner violence, but rather the perception of these experiences was responsible for partner violence among the LGBTQ college youth (21). The experiences of this study cannot be applied to understand the violence in presence of other internalized stressors, such as concealment of identity, internalized homophobia, and perceived stigma. Thus, there is a need to assess the relationship between perceived discrimination and IPV.

The association between IPV and discrimination can be studied using the Minority Stress Model, which provides an overview of the potential correlations between social stressors and negative health outcomes (22). This model suggests that individuals who identify with a minority group (i.e., bisexual and gay men) experience unique stressors that affect their physical and mental health (23). The Minority Stress Model asserts that internalized homophobia, perceived stigma, and discrimination are primary stressors sexual minority groups experience. Researchers have documented that these minority stress factors contribute to an increased risk of IPV among sexual minority populations (20, 21). For example, a study conducted in Atlanta, Georgia, documented a significant association between minority stress variables (i.e., internalized homophobia, lifetime experiences of racism, and homophobic discrimination) and physical, sexual, and emotional IPV among a sample of 1,075 bisexual and gay men (20).

The limited research posits that bisexual men regularly experience marginalization and stigmatization (1). Often, they are viewed as being confused, sexually promiscuous, immature, and engaged in polyamory (16). A stereotypical notion suggests bisexuality is a phase of false identity, making it difficult for these men to open up about their sexual orientation (16). Such discrimination and double-marginalization cause bisexual men to experience biphobia and binegativity (7). These few reasons possibly explain their reluctance to participate in IPV-related research studies (1). Owing to discrimination from both heterosexual and homosexual individuals, bisexual men may be at a greater risk of IPV victimization.

Given these findings, our study aims to fill the gap in the research literature on IPV experiences in bisexual and gay men. We examined the cross-sectional association between perceived discrimination and three forms of IPV in a sample of adult bisexual and gay men living in the U.S.

## 2. Methods

### 2.1. Study sample

We analyzed data from the Men’s Body Project (MBP), an online cross-sectional survey that examined various health behaviors and outcomes of bisexual and gay men living in the U.S. The health behaviors assessed in the MBP included, but are not limited to, dating, perceived discrimination, and intimate partner violence. Those eligible met the following criteria: (1) be living in the United States; (2) be at least 18 years of age but under 51 years; (3) self-identify as cis-gender gay or bisexual men; and (4) be English-speaking. The survey was administered in the spring of 2020 over a three-month period from March to May. All participants were recruited using Qualtrics Survey Panels and provided informed consent before completing the survey (24, 25). The study sample (N) comprised of 549 men.

### 2.2. Study ethics

The study was approved by the Institutional Review Board of the University of New Haven.

### 2.3. Measures

#### 2.3.1. Demographic variables

As part of the survey, participants provided their age (in years), ethnicity, race, sexual orientation identity, and current relationship status.

#### 2.3.2. Perceived discrimination

The perceived discrimination was measured using nine items from the Everyday Discrimination Scale (EDS) (26). The EDS is widely used in epidemiological literature to assess the subjective discriminatory experiences and unfair treatment in day-to-day life among marginalized populations (27). Study participants responded to nine items assessing experiences with chronic and routine unfair treatment in their everyday lives following an introductory prompt, “How often on a day-to-day basis do you experience each of the following types of discrimination?” The following items were included, (1) “you are treated with less courtesy than other people”; (2) “people act as if they are afraid of you”; (3) “you receive poorer service than other people at restaurants or stores”; (4) “people act as if you are not smart”; (5) “you are treated with less respect than other people are”; (6) “people act as if they think you are dishonest”; (7) “people act as if you are not as good as they are”; (8) “you are called names or insulted”; (9) “you are threatened or harassed.” The responses for each item were measured on a 4-point scale, ranging from 1 (often) to 4 (never), and added to create a range of scores from 9 to 36. A higher score represented greater experiences of perceived discrimination (26).

#### 2.3.3. Intimate partner violence

Our survey included three adapted items from the Centers for Disease Control and Prevention’s Youth Risk Behavior Surveillance System assessing experiences of intimate partner violence. They included: (1) “During the past 12 months, did your boyfriend, girlfriend, or partner ever hit, slap, or physically hurt you on purpose?” (Physical IPV); (2) “Have you ever been forced to have sexual intercourse when you did not want to?” (Non-physical coercion); (3) “During the past 12 months, how many times did anyone force you to do sexual things that you did not want to do (Count such things as kissing, touching, or being physically forced to have sexual intercourse)?” (Sexual IPV). Response options for the first two items were binary (yes/no). The options for the third item (Sexual IPV) were “0 times,” “1 time,” “2 or 3 times,” “4 or 5 times,” and “6 or more times” (28).

### 2.4. Statistical analysis

Descriptive statistics including means, standard deviations, and frequencies were calculated for sociodemographic variables, perceived discrimination scores, and the three forms of IPV. Using Chi-square (*χ*^2^ test) for categorical variables and student’s *t*-tests for continuous variables, we compared the distribution of the demographic variables across sexual orientation identity (bisexual men vs. gay men). Due to the small sample size in some of the strata, we dichotomized the variable of Sexual IPV so that responses of “0 times” were coded as 0 and 1 or more times was coded as 1. We then assessed the association between the participants’ perceived discrimination scores and the three forms of IPV using multivariable logistic regression. In our partially adjusted models, we controlled for demographic variables. The partially adjusted model use predictors like age, category, ethnicity, sexual orientation, race, relationship status, and employment. We additionally adjusted for perceived discrimination scores along with these predictors in our fully-adjusted models. The statistical significance was set at a *value of p* <0.05.

## 3. Results

Table 1 describes the sociodemographic characteristics of the sample. Of 549 participants, 48% (*n* = 263) identified as bisexual men and 52% (*n* = 286) as gay men. The majority of participants (54.6%) were 35–50 years of age, followed by those between 25–34 years (25.9%), and 18–24 years (19.5%), respectively. The age group distribution between bisexual and gay men was statistically significant, as more gay male respondents identified in the 35–50-year age group (65.4%) than those who identified as bisexual men (43.0%). Approximately 19% of the sample were Hispanic. The majority of respondents identified as White (71.4%, *n* = 392), followed by Black/African American (13.8%, *n* = 76), Asian/Pacific Islander (8.0%, *n* = 44), and American Indian/Other (6.7%, *n* = 37). Regarding relationship status, most men classified themselves as single/dating (55.9%, *n* = 307). There were significant differences in the distribution of relationship status between bisexual and gay men. Furthermore, bisexual men reported significantly higher perceived discrimination scores (higher by 3.4 points) when compared to their gay male counterparts in this study.

**Table 1:** Sociodemographic characteristics of study sample.

Characteristics	Gay (*n* = 286; 52.09%)	Bisexual (*n* = 263; 47.91%)	Total (*N* = 549)	Test statistic (*p*-value)
Age (years)				
18–24	39 (13.6)	68 (25.9)	107 (19.5)	28.61 (<0.001***)
25–34	60 (21.0)	82 (31.2)	142 (25.9)	
35–50	187 (65.4)	113 (43.0)	300 (54.6)	
Ethnicity				
Non-hispanic	227 (79.3)	219 (83.2)	446 (81.2)	
Hispanic	59 (20.6)	44 (16.7)	103 (18.8)	1.12 (0.28)
Race				
White	207 (72.4)	185 (70.3)	392 (71.4)	2.16 (0.53)
Black/African American	41 (14.3)	35 (13.3)	76 (13.8)	
Asian/Pacific Islander	23 (8.0)	21 (8.0)	44 (8.0)	
American Indian/other	15 (5.2)	22 (8.4)	37 (6.7)	
Relationship status				
Single/dating	174 (60.8)	133 (50.6)	307 (55.9)	45.54 (<0.001***)
Living with a partner	60 (21.0)	20 (7.6)	80 (14.6)	
Married/engaged	43 (15.0)	94 (35.7)	137 (25.0)	
Divorced/widowed/separated/other	9 (3.1)	16 (6.1)	25 (4.6)	
Work status				
Full-time	188 (65.7)	159 (60.5)	347 (63.2)	2.99 (0.56)
Part-time	26 (9.0)	23 (8.7)	49 (8.9)	
Student	22 (7.6)	29 (11.0)	51 (9.2)	
Unemployed	38 (13.3)	42 (16.0)	80 (14.6)	
Other	12 (4.2)	10 (3.8)	22 (4.0)	
Perceived discrimination	16.8 (7.2)	20.2 (7.9)	18.4 (7.7)	−5.22 (<0.001***)

*p-value < 0.05; **p-value < 0.01; ***p-value < 0.001.

Chi-square (*χ*^2^) and Student’s *t*-tests were used to compare differences across categorical and continuous variables between gay and bisexual men, respectively. SD, standard deviation. Results of Perceived Discrimination are presented as the mean and SD.

The prevalence of the three forms of IPV – Physical IPV, Non-physical coercion, Sexual IPV – are presented in Table 2. Sexual IPV (22.2%) was the most prevalent among the sample followed by Non-physical coercion (21.9%) and Physical IPV (16.4%). More bisexual men (21.3, 25.9, 30.0%) reported experiencing Physical IPV, Non-physical coercion, and Sexual IPV than gay men (11.9, 18.2, 15%) respectively. Participant characteristics were separated by IPV to highlight possible variables for further analysis (i.e., variable selection; see Supplementary Table S1).

**Table 2:** Prevalence of intimate partner violence in study sample.

Characteristics	Gay (*n* = 286)	Bisexual (*n* = 263)	Total (*N* = 549)	Test statistic (*p*-value)
During the past 12 months, did your boyfriend, girlfriend, or partner ever hit, slap, or physically hurt you on purpose? (Physical IPV)	34 (11.9)	56 (21.3)	90 (16.4)	8.17 (0.004**)
Have you ever been forced to have sexual intercourse when you did not want to? (Non-physical coercion)	52 (18.2)	68 (25.9)	120 (21.9)	4.28 (0.04*)
During the past 12 months, how many times did anyone force you to do sexual things that you did not want to do (Count such things as kissing, touching, or being physically forced to have sexual intercourse)? (Sexual IPV)^a^	43 (15.0)	79 (30.0)	122 (22.2)	16.99 (<0.001***)

*p-value < 0.05; **p-value < 0.01; ***p-value < 0.001.

^a^Item was dichotomized into a binary outcome.

Chi-square (*χ*^2^) were used to compare differences across categorical and continuous variables between gay and bisexual men, respectively.

Table 3 describes the partially-adjusted odds ratios (OR). Results show that bisexual men had 1.82 times the odds (95% CI: 1.12, 2.95) of Sexual IPV compared to gay men, after adjusting for demographic characteristics. There were no significant differences between Physical IPV and Non-physical coercion across sexual orientation. Participants between the ages of 25–34 years had 2.07 times the odds of experiencing Sexual IPV when compared to the two other age categories (95% CI: 1.05, 4.19). Furthermore, compared to single/dating participants, respondents who were married/engaged demonstrated significantly elevated odds for all three forms of IPV: Physical IPV (OR: 4.28, 95% CI: 2.34, 7.98), Non-physical coercion (OR: 2.96, 95% CI: 1.75, 5.08), and Sexual IPV (OR: 4.41, 95% CI: 2.53, 7.78). Participants who worked part time had less odds of experiencing Physical IPV (0.224, 95% CI: 0.035, 0.791), while those who reported unemployment and other as their work status had less odds of experiencing Sexual IPV (0.436, 95% CI: 0.186, 0.929) and (0.11, 95% CI: 0.006, 0.614) respectively.

**Table 3:** Odds of intimate partner violence and 95% confidence intervals (partially-adjusted).

Characteristics	Physical IPV	Non-physical coercion	Sexual IPV
Age			
18–24 (ref)	1.00 (ref)	1.00 (ref)	1.00 (ref)
25–34	1.61 (0.74, 3.63)	1.18 (0.60, 2.32)	2.07 (1.05, 4.19)*
35–50	0.63 (0.28, 1.46)	0.68 (0.35, 1.33)	0.65 (0.32, 1.35)
Sexual orientation			
Gay (ref)	1.00 (ref)	1.00 (ref)	1.00 (ref)
Bisexual	1.32 (0.78, 2.24)	1.10 (0.69, 1.74)	1.82 (1.12, 2.95)*
Ethnicity			
Non-Hispanic (ref)	1.00 (ref)	1.00 (ref)	1.00 (ref)
Hispanic	0.86 (0.42, 1.68)	0.812 (0.43, 1.47)	1.43 (0.77, 2.62)
Race			
White (ref)	1.00 (ref)	1.00 (ref)	1.00 (ref)
Black/African American	1.31 (0.61, 2.69)	1.12 (0.58, 2.07)	1.75 (0.90, 3.32)
Asian/Pacific Islander	0.67 (0.21, 1.75)	0.63 (0.24, 1.44)	2.00 (0.90, 4.25)
Other/American Indian	0.91 (0.29, 2.52)	0.52 (0.16, 1.40)	0.44 (0.14, 1.19)
Relationship status			
Single/Dating (ref)	1.00 (ref)	1.00 (ref)	1.00 (ref)
Living with a partner	1.25 (0.51, 2.79)	0.759 (0.34, 1.55)	0.976 (0.42, 2.08)
Married/Engaged	4.28 (2.34, 7.98)***	2.96 (1.75, 5.08)***	4.41 (2.53, 7.78)***
Divorced/widowed/separated/other	2.77 (0.83, 7.99)	1.78 (0.60, 4.65)	1.87 (0.56, 5.36)
Work status			
Full time (ref)	1.00 (ref)	1.00 (ref)	1.00 (ref)
Part time	0.22 (0.03, 0.79)*	0.96 (0.40, 2.09)	1.43 (0.63, 3.05)
Student	0.80 (0.26, 2.12)	0.80 (0.31, 1.90)	1.17 (0.49, 2.68)
Unemployed	0.84 (0.38, 1.73)	1.50 (0.80, 2.72)	0.43 (0.18, 0.92)*
Other	0.19 (0.01, 1.03)	0.36 (0.05, 1.37)	0.11 (0.00, 0.61)*

*p-value < 0.05; **p-value < 0.01; ***p-value < 0.001.

Models adjusted for age, sexual orientation, ethnicity, race, relationship status, and work status.

Results from our models adjusting for demographic characteristics and perceived discrimination scores (fully-adjusted) are illustrated in Table 4. After additionally adjusting for perceived discrimination scores, the association between sexual orientation and Sexual IPV documented in the partially-adjusted analyses was no longer significant. However, participants ages 25–34 years old still had significantly elevated odds of experiencing Sexual IPV (2.22, 95% CI: 1.06, 4.77). In addition, compared to their single/dating counterparts, married/engaged participants also demonstrated elevated odds for Physical IPV (OR: 2.14, 95% CI: 1.04, 4.38) and Sexual IPV (OR: 2.44, 95% CI: 1.29, 4.60). Participants who reported being divorced, widowed, separated, or other also had significantly higher odds of experiencing Physical IPV (4.03, 95% CI: 1.04, 13.94) after adjustment. Among the racial groups, individuals in the American Indian/Other category demonstrated significantly lower odds of Sexual IPV (OR: 0.303, 95% CI: 0.093, 0.871) compared to their White counterparts. Participants working part time continued to have less odds of experiencing Physical IPV (0.198, 95% CI: 0.03, 0.742), and unemployed participants also continued to have lower odds of experiencing Sexual IPV (0.374, 95% CI: 0.148, 0.859). Participants who reported other as their work status, however, continued to have less odds of experiencing Sexual IPV (0.05, 95% CI: 0.00, 0.39). Perceived discrimination scores were significantly associated with elevated odds for Physical IPV (OR: 1.18, 95% CI: 1.13, 1.24), Non-physical coercion (OR: 1.15, 95% CI: 1.11, 1.19), and Sexual IPV (OR: 1.16, 95% CI: 1.12, 1.20).

**Table 4:** Odds of intimate partner violence and 95% confidence intervals (fully-adjusted).

Characteristics	Physical IPV	Non-physical coercion	Sexual IPV
Age (years)			
18–24 (ref)	1.00 (ref)	1.00 (ref)	1.00 (ref)
25–34	1.95 (0.84, 4.66)	1.23 (0.60, 2.54)	2.22 (1.06, 4.77)*
35–50	0.98 (0.42, 2.38)	0.91 (0.46, 1.86)	0.82 (0.38, 1.76)
Sexual orientation			
Gay (ref)	1.00 (ref)	1.00 (ref)	1.00 (ref)
Bisexual	0.97 (0.53, 1.77)	0.83 (0.50, 1.38)	1.51 (0.89, 2.56)
Ethnicity			
Non-hispanic (ref)	1.00 (ref)	1.00 (ref)	1.00 (ref)
Hispanic	1.12 (0.51, 2.34)	0.92 (0.46, 1.77)	1.79 (0.91, 3.46)
Race			
White (ref)	1.00 (ref)	1.00 (ref)	1.00 (ref)
Black/African American	0.95 (0.41, 2.09)	0.83 (0.41, 1.62)	1.35 (0.66, 2.69)
Asian/Pacific Islander	0.53 (0.15, 1.54)	0.50 (0.17, 1.26)	1.91 (0.79, 4.46)
Other/American Indian	0.66 (0.19, 2.00)	0.35 (0.10, 1.02)	0.30 (0.09, 0.87)*
Relationship status			
Single/dating (ref)	1.00 (ref)	1.00 (ref)	1.00 (ref)
Living with a partner	1.64 (0.63, 3.93)	0.83 (0.36, 1.80)	1.06 (0.43, 2.41)
Married/engaged	2.14 (1.04, 4.38)*	1.65 (0.89, 3.03)	2.44 (1.29, 4.60)**
Divorced/widowed/separated/other	4.03 (1.04, 13.94)*	2.13 (0.65, 6.28)	2.09 (0.52, 7.27)
Work status			
Full time	1.00 (ref)	1.00 (ref)	1.00 (ref)
Part time	0.19 (0.03, 0.74)*	0.94 (0.38, 2.14)	1.46 (0.62, 3.26)
Student	0.77 (0.24, 2.17)	0.74 (0.27, 1.85)	1.00 (0.39, 2.45)
Unemployed	0.76 (0.32, 1.72)	1.56 (0.79, 3.01)	0.37 (0.14, 0.85)*
Other	0.09 (0.00, 0.70)	0.28 (0.03, 1.27)	0.05 (0.00, 0.39)*
Perceived discrimination score	1.18 (1.13, 1.24)***	1.15 (1.11, 1.19)***	1.16 (1.12, 1.20)***

*p-value < 0.05; **p-value < 0.01; ***p-value < 0.001.

Models adjusted for age, sexual orientation, ethnicity, race, relationship status, work status, and perceived discrimination.

## 4. Discussion

Our study aimed to assess the association between the three forms of IPV and perceived discrimination among a sample of bisexual and gay men living in the U.S. A prevalence of 16.4, 21.9, and 22.2% were observed for Physical, Non-physical coercion, and Sexual forms of IPV. Guided by the Minority Stress Model, we specifically examined the association between IPV and participants’ perceived discrimination scores, which reflected their experiences with chronic, routine day-to-day unfair treatment. Results from our logistic regression analyses suggested a significant relationship between perceived discrimination and all three forms of IPV. Specifically, participants who reported higher perceived discrimination scores – or greater self-reported experiences with day-to-day unfair treatment – demonstrated elevated odds of IPV.

Our findings corroborate those of previous studies that documented a statistically significant association between Physical/Sexual IPV and discrimination. For instance, the results of a study conducted in Atlanta, Georgia by Stephenson and Finneran suggested a significant association between Physical/Sexual IPV and minority stress measures (i.e., internalized homophobia, racism, and homophobic discrimination) in bisexual and gay men (20). A study conducted by Bartholomew et al. (29) had highlighted that 44% bisexual and gay men in same-sex relationships reported physical IPV victimization. In their study, Stults et al. demonstrated that discrimination/internalized negative beliefs about being bisexual or gay contribute significantly to IPV among young men who have sex with men during early adulthood (30). This discrimination due to sexual orientation may be indicative of high prevalence of IPV.

Furthermore, our findings suggest a higher prevalence of IPV among bisexual men when compared with gay men. This is consistent with previous research showcasing the difference in IPV prevalence between the two sexual minorities (31, 32). Such findings also support previous calls to disaggregate sexual orientation identity groups as opposed to viewing them as a monolith for research purposes. In the absence of adequate support and validation from the LGB community, bisexual men often experience additional stress related to IPV (7). Second, bisexual men who are victims of IPV have also reported hiding their sexual identity (‘double – closeted’), due to the fear of being rejected from their family and friends (1). Third, bisexual men are subjected to discrimination, stigmatization, and double marginalization by both heterosexual and homosexual individuals. This phenomenon of double discrimination is referred to as biphobia. The maltreatment of bisexual men by both communities coupled with the disregard for the violence inflicted on the bisexual men may increase one’s risk of IPV. Furthermore, studies suggest biphobia within the LGB community increases bisexual men’s risk for IPV victimization while also reducing their access to help-giving resources (32, 33).

Our study suggests relationship status is crucial to experiencing intimate partner violence. The results show that divorced/separated/widowed and engaged/married sexual minority men are at significantly higher risk of physical and sexual IPV. In the past, research has documented similar results suggesting relationship status can be a risk factor for IPV. A study by Carvalho et al., observed that sexual minority adults in committed relationships were at higher risk of Physical IPV than their counterparts (19). This violence could be due to increased stress, financial constraints, and other factors arising from separation. Another study found that young sexual minority men in romantic relationships reported a greater likelihood of experiencing IPV. However, participants were not asked to specify the category of their relationship in which violence occurred (30). Given the limited existing studies assessing the correlation between IPV and relationship status, more research is needed to explore the factors that contribute to IPV based on relationship patterns among sexual minority men.

The framework of the Minority Stress Model posits that sexual orientation-based disparities in health outcomes exist because of unique and chronic stressors imposed upon sexual minorities (22). Such stressors include a variety of forms of discrimination, including homophobia and biphobia, that may result in associated coping mechanisms that have health consequences. In our study, we linked experiences of IPV with sexual minority men’s self-reported experiences with perceived discrimination, a potential indicator of these men’s chronic exposure to a form of stress that exists within their social and cultural structures. In particular, higher perceived discrimination scores were associated with elevated odds of the various forms of IPV in our sample of bisexual and gay men. Thus, it is imperative that the role of discrimination be further investigated through research, but also be adequately considered in existing interventions aimed at alleviating the burden in sexual minority men. As suggested in existing studies, a one-size-fits-all approach to IPV assessment and treatment is not effective, and falsely operates under the assumption that IPV experiences in heterosexual, homosexual, and bisexual relationships are universally similar (7). Homophobia and other discrimination toward bisexual and gay men are barriers to seeking treatment and support for IPV (7).

Our study has key limitations that need to be considered. The cross-sectional design of the online study prevents our ability to conclude causality and temporality between perceived discrimination and the three forms of IPV. In addition, our convenience sample was over-represented by White men; hence, our results cannot be generalized to all individuals who come from different racial and ethnic groups. Also, men older than 50 years were not included in this study, which neglects potential experiences with IPV among those living in older age groups. Future studies should be inclusive of a sample drawn from all ages and racial/ethnic groups of sexual minority men. Furthermore, excluding heterosexual men contributed to the limited understanding of the differences and/or similarities between perceived discrimination and IPV when compared with sexual minority men. Given experiences with IPV were self-reported, it is possible our prevalence of each form of IPV was under-reported due to participants’ unwillingness to respond to questions (20). In our study, emotional and psychological forms of IPV were not assessed.

Additionally, our study used the Everyday Discrimination Scale that captures the chronic and episodic features of interpersonal discrimination (26). However, even though the scale measures a crucial aspect of discrimination, it does not cover all the factors that may lead to discrimination based on sexual orientation. Studies have linked IPV experiences to race-related discrimination but not sexual orientation-identity-discrimination (34). Specifically, Robles et al. (34) found that race-based discrimination increased the odds of IPV victimization in Latino sexual minority men. Thus, additional studies are needed to elucidate the potential role of specific forms of discrimination sexually minority men face to better predict their risk of IPV victimization. Furthermore, there is also a possibility that variables such as education, the number and gender of the sexual partners, not measured in the present study, may confound the association between perceived discrimination and IPV. Thus, we cannot ascertain whether experiences of intimate partner violence were perpetrated by an intimate or a non-intimate partner. Another limitation was that items used to measure intimate partner violence in adult populations were taken from the Youth Risk Behavior Surveillance System. Future research should consider utilizing scales and other assessments specifically designed to capture experiences and additional elements of IPV (e.g., controlling behaviors), including IPV in sexual minority men (8, 35). Our study’s item assessing Physical IPV, for example, implied participants were in monogamous relationships, which may not comprehensively capture such experiences among those with multiple partners. Finally, as a result of the COVID-19 pandemic, which began around the same time as the study’s implementation, response rates may have been impacted. Considering the Minority Stress Model, future studies will need to comprehensively assess additional forms of discrimination and related stressors experienced by sexual minority men, such as internalized homophobia and stigma consciousness.

The study presented several notable strengths. This study enabled us to assess the association between IPV and perceived discrimination among sexual minority men, an area that has been under-researched (36). Our results are congruent with prior qualitative study findings indicating the urgent need to address this issue among bisexual and gay men (37). Furthermore, a significant strength of our research was our ability to draw comparisons between gay and bisexual men. Bisexual men represent a sexual orientation identity group that remains significantly understudied in the IPV literature and has historically been combined with gay men and other sexual minorities for research purposes. We believe our findings may help to fill the existing literature gap and draw future recommendations for tackling and alleviating IPV issues among sexual minority men in the U.S.

Given these findings, reducing minority stress in sexual minority men must be made a priority, as it continues to be a risk factor for IPV victimization (13). More longitudinal studies are needed to investigate the causes of IPV and discrimination so that support and resources can be made available to the affected victims. Previous studies have primarily focused on IPV among heterosexual groups; thus, increasing the need to study the origin, consequences, and preventive strategies to reduce IPV prevalence among sexual minority men (38, 39). Additionally, researchers must adopt qualitative approaches for identifying the potential IPV risk factors surrounding these marginalized groups. Elucidating victims’ narratives on their experiences with IPV and perceptions toward seeking support may assist in tailoring effective intervention strategies, formulate policies, and legislation. Considering that our study found an increased prevalence of IPV in bisexual men, it is imperative that clinicians and other healthcare providers begin to tailor specific services toward sexual minority men, much like the work done for female IPV victims.

Policies that aim to tackle stigma and discrimination among the LGBTQ+ groups at the community (e.g., pro-acceptance campaigns) and structural level (e.g., anti-conversion therapy laws) can help foster a healthy family environment, which may be beneficial in preventing and reducing IPV among sexual minority men (40). Provision of education and training to health care workers to facilitate screening of IPV among bisexual and gay men will be crucial in providing counseling and preventive care to affected individuals (35). Additionally, hate crimes and criminal justice laws are required to prevent violence and discrimination toward sexual minority men. According to the State Equality Index, a total of 23 states have laws to address hate and crimes based on sexual orientation and gender identity, whereas only 11 states address crimes based on only sexual orientation (41). We believe more states need to adopt laws that target stigma and discrimination among sexual minority groups. These laws can ensure that the prevalence of IPV among sexual minority men is reduced in the nearby future.

## 5. Conclusion

In recent years, IPV among sexual minority men have received considerable attention from researchers worldwide, including the United States. To understand the complexities of IPV, concentrated efforts to identify the causes of violence are required. Additionally, educational campaigns that address myths about bisexuality and facilitate help-seeking behavior should be a priority to fight discrimination. Such educational efforts should also aim to address myths surrounding IPV experiences in diverse samples of cis-gender men, as research in this area has widely focused on cis-gender women (42). Considering the framework of the Minority Stress Model, future studies examining the psychosocial mechanisms linking discrimination to IPV are warranted. Furthermore, social policies aimed at preventing discrimination based on sexual orientation identity may also remove barriers to these men in seeking IPV-related support services. Healthcare providers and other professionals should consider deriving and implementing efforts – from policies to intervention strategies – to remove barriers sexual minority men face when accessing IPV-related support.

## Data availability statement

The raw data supporting the conclusions of this article will be made available by the authors upon request.

## Ethics statement

The studies involving human participants were reviewed and approved by the University of New Haven Institutional Review Board (#2020–015). The patients/participants provided their written informed consent to participate in this study.

## Author contributions

KR, LJ, and AT wrote the original article. NB analyzed the data. AT provided the suggestions on data analysis and revising the paper. AT supervised the all aspects of the research and contributed to writing the article. All authors critically interpreted the findings and edited the article.

## Conflict of interest

The authors declare that the research was conducted in the absence of any commercial or financial relationships that could be construed as a potential conflict of interest.

## Publisher’s note

All claims expressed in this article are solely those of the authors and do not necessarily represent those of their affiliated organizations, or those of the publisher, the editors and the reviewers. Any product that may be evaluated in this article, or claim that may be made by its manufacturer, is not guaranteed or endorsed by the publisher.
