# Supplementary material for: Discrimination and intimate partner violence among a sample of bisexual and gay men in the United States: a cross-sectional study
Source: Front Public Health. 2023 Jul 31;11:1182263. doi: 10.3389/fpubh.2023.1182263 (PMC10423812; doi:10.3389/fpubh.2023.1182263)
Supplement: Supplementary file 1 [file Table_1.docx]

**Supplementary Table 1**

*Participant characteristics stratified by intimate partner violence (IPV).*

| Characteristic | Physical IPV | Non-physical coercion | Sexual IPV | |
| --- | --- | --- | --- | --- |
| Age (years) |  |  | |  |
| 18-24 | 13 (12.1) | 21 (19.6) | | 22 (20.4) |
| 25-34 | 39 (27.5) | 42 (29.6) | | 53 (37.3) |
| 35-50  Test statistic (p-value) | 38 (12.7)  17.15 (0.002**) | 57 (19.0)  6.70 (0.03*) | | 47 (15.7)  26.4 (<0.001***) |
| Ethnicity |  |  | |  |
| Non-Hispanic | 73 (16.4) | 101 (22.6) | | 96 (21.5) |
| Hispanic  Test statistic (p-value) | 17 (16.5)  0.00 (1.00) | 19 (18.4)  0.64 (0.42) | | 26 (25.2)  0.47 (0.49) |
| Race |  |  | |  |
| White | 66 (16.8) | 90 (23.0) | | 81 (20.7) |
| Black/African American | 13 (17.1) | 18 (23.7) | | 21 (27.6) |
| Asian/Pacific Islander | 5 (11.4) | 7 (15.9) | | 14 (31.8) |
| American Indian/Other  Test statistic (p-value) | 6 (16.2)  0.90 (0.82) | 5 (13.5)  2.85 (0.41) | | 6 (16.2)  4.95 (0.17) |
| Relationship Status |  |  | |  |
| Single/Dating | 30 (9.7) | 54 (17.6) | | 47 (15.3) |
| Living with a partner | 9 (11.3) | 10 (12.5) | | 10 (12.5) |
| Married/Engaged | 46 (33.6) | 50 (36.5) | | 60 (43.8) |
| Divorced/Widowed/Separated/  Other  Test statistic (p-value) | 5 (20.0)  NA^†^ (<0.001***) | 6 (24.0)  24.6 (<0.001***) | | 5 (20.0)  49.8 (<0.001***) |
| Work Status |  |  | |  |
| Full-time | 70 (20.2) | 80 (23.1) | | 88 (25.4) |
| Part-time | 2 (4.0) | 9 (18.4) | | 12 (24.5) |
| Student | 6 (11.8) | 8 (15.7) | | 12 (23.5) |
| Unemployed  Other  Test statistic (p-value) | 11 (13.8)  1 (4.5)  NA^†^ (0.011*) | 21 (26.3)  2 (9.0)  NA^†^ (0.34) | | 9 (11.3)  1 (4.5)  NA^†^ (0.013*) |
| Perceived Discrimination Score (Mean for Yes and No group)  Test statistic (p-value) | Yes = 26.1  No =16.9  11.6 (<0.001***) | Yes = 24.6  No = 16.7  10.7 (<0.001***) | | Yes = 25.0  No = 16.5  12.7 (<0.001***) |

* *p-*value < 0.05; ** *p-*value < 0.01; *** *p-*value < 0.001

Note: Chi-square (χ^2^) and Student’s *t-*tests were used to compare differences across categorical and continuous variables between gay and bisexual men, respectively. NA = Not available. ^†^ Indicates Fisher’s exact test used when expected cell count was <5.
